# The Effects of Botulinum Toxin on Sleep Bruxism: An Electromyographic Study with the Portable Bruxoff Holter System

**DOI:** 10.3390/jcm15093275

**Published:** 2026-04-25

**Authors:** Mohammad Farazpey, Vincenzo Bellitto, Giovanna Ricci, Giulio Nittari

**Affiliations:** 1 Smiles Dental Clinic, 10141 Turin, Italy; dr.farazpey@yahoo.it; 2 School of Medicinal and Health Products Sciences, University of Camerino, 62032 Camerino, Italy; vincenzo.bellitto@unicam.it; 3 Section of Legal Medicine, School of Law, University of Camerino, 62032 Camerino, Italy; giovanna.ricci@unicam.it

**Keywords:** sleep bruxism, electromyography, botulinum toxin, Bruxoff system

## Abstract

**Background:** Sleep bruxism involves repetitive jaw-muscle activity, including teeth clenching, grinding, or mandibular bracing. Despite the growing interest in botulinum toxin type A (BTX-A) as a therapeutic intervention for bruxism, evidence remains limited, particularly regarding studies using portable electromyography (EMG) monitoring devices. This study evaluated the effects of BTX-A injections into the masseter muscle on the reduction of bruxism activity, as measured using the portable electromyographic Holter Bruxoff system. **Methods:** Adult patients with diagnosed sleep bruxism were monitored for two nights using the Bruxoff device to record masseter EMG activity, respiratory rate, and heart rate. After receiving standardized bilateral masseter BTX-A injections, participants underwent the same monitoring protocol 40 days later. Statistical analyses compared pre- and post-treatment values, and effect sizes were calculated. **Results:** Ten participants (60% women; mean age 47.6 ± 4.4 years) completed the study. The Bruxism Index showed a marked reduction, dropping from 12.2 ± 1.32 at baseline to 7.4 ± 1.35 after 40 days, a statistically significant change (t (9) = 10.23, *p* < 0.001; Cohen’s d = 3.25). Average heart rate also decreased significantly, from 64.4 ± 2.99 to 62.6 ± 2.63 (t (9) = 2.86, *p* = 0.018; Cohen’s d = 0.91). However, the respiratory rate measurement remains stable. **Conclusions:** BTX-A injections into the masseter muscles produced a marked reduction in sleep-related bruxism activity as measured by portable EMG. These findings support BTX-A as a promising and effective treatment option for sleep bruxism.

## 1. Introduction

Bruxism is characterized as a repetitive activity of the jaw musculature. Sleep bruxism (SB) specifically involves involuntary clenching or grinding of the teeth and/or increased activity of the masticatory muscles during sleep [1]. It is becoming one of the most frequently encountered conditions in dental practice. Its rising prevalence is often associated with everyday parafunctional habits that place additional strain on the masticatory system, a pattern that may be further intensified by psychological stress [1,2,3]. Its prevalence in adults is estimated to range from 8% to 15%, and the condition can significantly affect both oral health and overall quality of life [4]. Clinically, sleep bruxism may lead to tooth wear or fractures, periodontal damage, temporomandibular joint disorders, orofacial pain, and sleep disturbances that can impact not only affected individuals but also their bed partners [5,6].

Bruxism does not typically arise from a single identifiable cause; instead, it is understood as a condition with a multifactorial origin [7]. Its pathophysiology involves an interplay of central, peripheral, and psychosocial factors. Central mechanisms include alterations in neurotransmitter activity and the presence of sleep-related arousal micro-events, both of which can trigger or amplify masticatory muscle activity [8]. Peripheral contributors, such as malocclusion or dental interferences, may influence the pattern or intensity of bruxing behavior, although they are not considered primary causes [3]. Psychosocial factors, particularly stress and anxiety, are also recognized as significant risk elements that can exacerbate the condition [4]. Electromyographic studies consistently demonstrate heightened activity of the masseter and temporalis muscles in individuals with bruxism, with these episodes frequently aligning with specific sleep stages [9]. Although no definitive cure for bruxism currently exists, its symptoms can be effectively managed, and the likelihood of complications reduced [10]. When the condition is strongly influenced by psychological distress, professional support, such as stress-management strategies or psychological counselling, may be beneficial in addressing underlying emotional factors [11].

The conventional treatment for bruxism consists of a combination of therapeutic approaches designed to reduce symptoms and prevent dental or muscular complications. A number of strategies have been employed to protect the dentition, including occlusal splints or night guards, stress reduction techniques, behavioral therapy, pharmacological agents such as muscle relaxants or anxiolytics to modulate muscle activity, and physical therapy to enhance neuromuscular function [10,11,12]. However, although these interventions are widely used, they often provide only partial symptom relief, are highly dependent on patient adherence, and may not yield sustained long-term results.

As clinical experience has shown, occlusal splints alone do not always provide sufficient symptom control in patients with bruxism, prompting the use of adjunctive therapies such as botulinum toxin type A (BTX-A). BTX-A, a neurotoxin derived from *Clostridium botulinum*, induces temporary muscle relaxation by inhibiting acetylcholine release at the neuromuscular junction. Its therapeutic applications are well established in neurology, aesthetic medicine, and pain management. In the context of bruxism, targeted injection of BTX-A into the masseter muscle has been reported to decrease muscle overactivity, alleviate associated symptoms such as morning headaches, and contribute to the reduction of masseteric hypertrophy when used alongside conventional splint therapy [12,13]. BTX-A has recently emerged as a promising treatment for sleep bruxism. It reduces the hyperactivity of the masseter muscle, which may result in a decrease in nocturnal grinding and clenching.

Despite growing interest in the use of BTX-A for managing bruxism, the current body of evidence remains limited, especially regarding studies that incorporate objective electromyographic (EMG) assessment with portable monitoring technologies. To address this gap, the present investigation utilizes the Bruxoff Holter system, a validated, portable EMG device capable of capturing bruxism episodes alongside cardiorespiratory parameters during sleep, to provide more robust and clinically relevant data.

Therefore, the primary aim of this study is to determine the effects of BTX-A injections into the masseter muscle on the bruxism index measured by the Holter Bruxoff system. Secondary objectives include examining treatment-related changes in masseter EMG activity, respiratory patterns, heart rate, and sleep duration before and after treatment. It is hypothesized that intramuscular BTX-A administration will significantly reduce bruxism activity, as indicated by a measurable decrease in EMG-detected bruxism episodes.

## 2. Materials and Methods

### 2.1. Study Design, Participants, and Intervention

In this study, we employed a prospective experimental design with pre- and post-treatment assessments, which allowed within-subject comparisons of bruxism activity before and after treatment. The study was conducted at a Private practice in the Province of Turin among patients with suspected bruxism.

The trial of the study began on 15 October 2022 and continued until 24 January 2024, a period during which Bruxoff was applied to the patients included in the study.

In this study, ten adult patients diagnosed with sleep bruxism were recruited. This study was designed as a pilot study to evaluate the preliminary efficacy of botulinum toxin in adults with sleep bruxism. No formal sample size calculation was performed, as the primary aim was to assess efficacy and gather preliminary data for future larger-scale studies. Participants were eligible for inclusion if they were between 30 and 55 years of age and met the diagnostic criteria for sleep bruxism. Diagnosis required the presence of clinical indicators, such as dental wear or masseter hypertrophy, and confirmation of bruxism activity through electromyographic assessment using the Bruxoff device. Exclusion criteria included diagnosed neuromuscular disorders, previous administration of botulinum toxin type A, pregnancy, or any contraindications to botulinum toxin therapy

**Figure 1:**
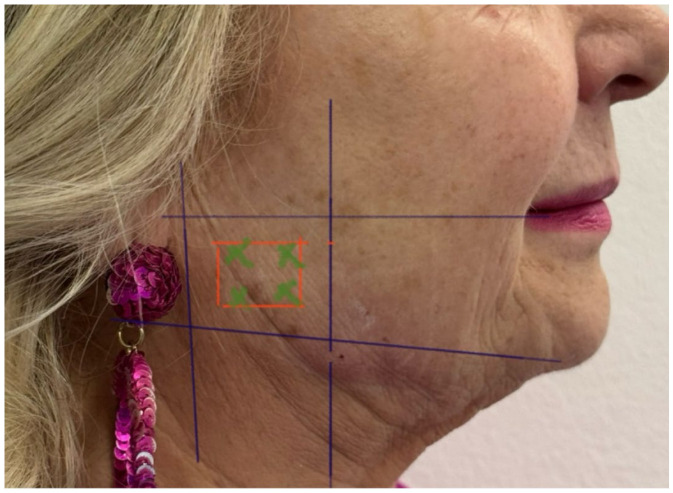
The safety Zone.

After baseline monitoring, participants underwent standardized intramuscular administration of botulinum toxin type A (BTX-A) into both masseter muscles. Each patient received 6 units of BTX-A, prepared from a 50-unit vial of Bocouture powder reconstituted with 1.25 mL of 0.9% saline. In other words, BTX-A was injected into the masseter muscles at four points within the established “safety zone” of each muscle. Each injection point received 6 units, for a total of 24 units per masseter. This dosing protocol was selected based on clinical experience to balance efficacy with minimal risk of adverse effects. The protocol used four injection points per masseter. Guided by established anatomical landmarks and adjacent critical structures, injections were confined to the recommended “safety zone,” defined as the area inferior to an imaginary line extending from the earlobe to the oral commissure and located between the palpable posterior border of the masseter and a point approximately 1 cm posterior to its palpable anterior border. Furthermore, Figure 1 illustrates the designated safety zone, outlining the anatomical boundaries recommended for the administration of BTX-A (Figure 1).

Data collection included baseline and follow-up monitoring with the Bruxoff Holter device. Before treatment, participants completed two consecutive nights of baseline recording, during which the device measured masseter electromyographic (EMG) activity, the bruxism index (episodes per hour), heart rate, and respiratory rate. At 40 days post-intervention, the same protocol was repeated over two consecutive nights under identical conditions to allow direct comparison with baseline measurements.

Outcome measures focused primarily on changes in the bruxism index from pre- to post-treatment, serving as the primary indicator of therapeutic efficacy. Secondary outcomes included changes in masseter EMG activity, heart rate, and respiratory rate, allowing for a broader assessment of the physiological responses associated with the intervention.

### 2.2. Statistical Analysis

Descriptive statistics were computed to characterise the sample’s demographic variables, including age. Prior to conducting the main analyses, data were assessed for normality. Results from the Shapiro–Wilk tests indicated that the primary outcome (Bruxism Index), as well as the secondary outcomes of heart rate and respiratory rate, met the assumptions of normal distribution. Conversely, sleep duration violated normality assumptions. Accordingly, paired *t*-tests were employed for variables exhibiting normal distributions, whereas the Wilcoxon signed-rank test was used for non-normally distributed variables to evaluate pre- to post-treatment changes in bruxism activity. Data are reported as means and standard deviations. The magnitude of treatment effects was quantified using Cohen’s *d*, interpreted according to conventional thresholds for small (0.2), medium (0.5), and large (0.8) effects. All statistical analyses were performed using IBM SPSS Statistics for Windows, version 25.0 (IBM Corp., Armonk, NY, USA).

## 3. Results

**Figure 2:**
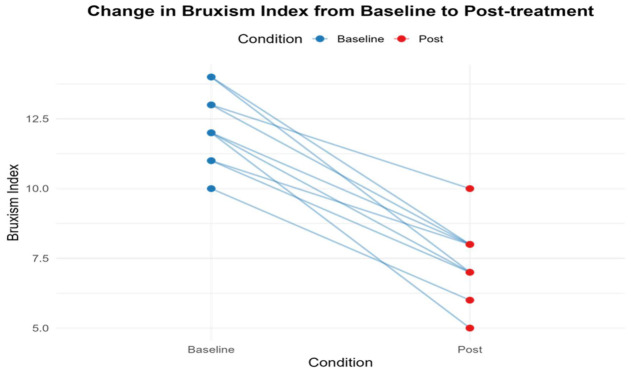
Individual changes in the Bruxism Index before and after treatment.

A total of ten patients (60% women) with a mean age of 47.6 ± 4.4 years (range: 42–55) completed the study. The BTX-A injections were well tolerated, with no major adverse events observed. Two participants reported mild, transient soreness at the injection site, which resolved spontaneously. All participants had a diagnosis of sleep bruxism and underwent assessments both at baseline and after treatment. The average Bruxism Index decreased from 12.2 ± 1.32 at baseline to 7.4 ± 1.35 after 40 days of treatment. Our analysis revealed a statistically significant reduction [t (9) = 10.23, *p* < 0.001, Cohen’s d = 3.25, indicating a large effect. Additionally, the participants’ mean heart rate declined from 64.4 ± 2.99 to 62.6 ± 2.63 over the same period, with paired *t*-test analysis confirming a significant decrease [t (9) = 2.86, *p* = 0.018], Cohen’s d = 0.91, indicating a large effect. These findings suggest that intramuscular BTX-A injections into the masseter muscle effectively reduce bruxism activity. However, respiratory rate and sleep duration did not demonstrate significant changes following treatment (Table 1). In addition, Figure 2 illustrates the individual changes in the Bruxism Index before and after treatment.

**Figure 3:**
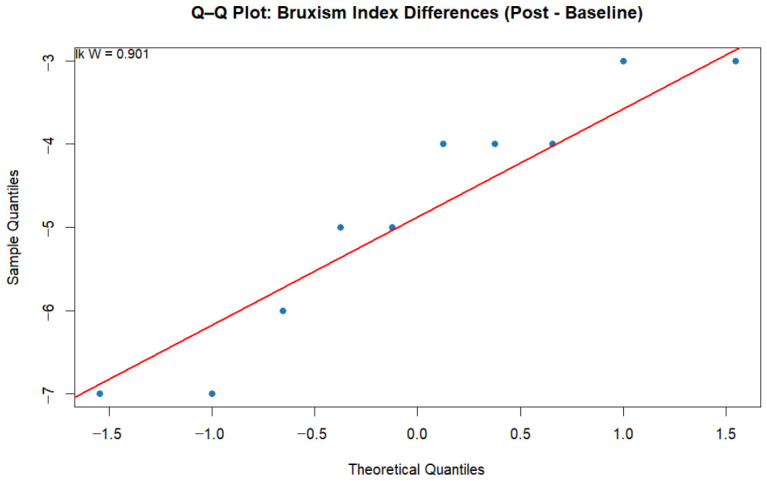
The Q-Q plot of normality of difference scores (Post–Baseline) for the Bruxism Index.

Normality of the difference scores for all outcome variables was tested using the Shapiro–Wilk test. The Bruxism Index (*W* = 0.901, *p* = 0.225), heart rate (*W* = 0.955, *p* = 0.632), and respiratory rate (*W* = 0.928, *p* = 0.384) showed no significant departures from normality. However, sleep duration deviated slightly from normality (*W* = 0.810, *p* = 0.041 *). Additionally, Figure 3 provides a visual assessment of the Bruxism Index through Q–Q plots, which further support the assumption of normality.

## 4. Discussion

The findings of the present study indicate that intramuscular administration of BTX-A into the masseter muscle produces a significant reduction in bruxism activity, as quantified through portable electromyographic monitoring with the Holter Bruxoff system. A substantial decrease in the mean Bruxism Index was observed 40 days after treatment, reflecting a pronounced attenuation of muscle activity associated with sleep bruxism. Moreover, the magnitude of change demonstrated a large effect size, underscoring the robustness of the therapeutic response.

In addition to the primary outcome, notable changes were detected in one of the secondary physiological parameters. Participants exhibited a reduction in mean heart rate over the same period, and this decrease also corresponded to a large effect size, suggesting that BTX-A treatment may exert broader physiological influences beyond localized muscle relaxation. These observations collectively support the efficacy of BTX-A injections in mitigating sleep bruxism-related muscular hyperactivity. Although heart rate changes are often observed during bruxism episodes, they are part of the autonomic response associated with the event rather than a direct result of bruxism. Heart rate is also affected by multiple factors, including lifestyle, stress, and comorbid conditions that were not controlled in this study. Therefore, these measures should be interpreted with caution and cannot serve as a specific marker of bruxism severity. Nonetheless, heart rate monitoring offered exploratory insight into the physiological response occurring alongside bruxism episodes. Future studies with larger cohorts and controlled conditions are necessary to better understand this relationship. Conversely, no significant alterations were identified in respiratory rate or total sleep duration following treatment, indicating that the intervention’s effects may be more specific to neuromuscular activity rather than global sleep architecture or autonomic regulation.

These findings are consistent with those of previously published clinical trials demonstrating that the administration of BTX-A significantly reduces electromyographic activity and improves symptoms associated with bruxism. Previous studies have repeatedly shown that weakening the masseter muscle with BTX-A reduces the intensity and frequency of jaw-muscle contractions during sleep [13,14,15], and our findings support these findings. In this study, a portable Holter-based EMG device was used to monitor bruxism activity over a period of multiple nights in participants’ natural sleeping environments. It offers a methodological advantage over studies that rely exclusively on subjective symptom reports, clinician impressions, or single-night laboratory assessments, which may be influenced by night-to-night variability or first-night effects. This study provides a more stable and ecologically valid representation of sleep bruxism patterns by capturing repeated measurements across several nights, thus enhancing the reliability and interpretation of the observed treatment effects.

It is evident from this study that BTXA may be an effective therapeutic alternative for individuals with refractory bruxism, particularly those who have demonstrated limited or no improvement with conventional approaches such as occlusal splints, behavioral interventions, or stress management strategies. Attenuating excessive masseter muscle activity, BTXA offers a targeted mechanism of action that directly addresses the muscular component of bruxism rather than relying only on behavioral modification or mechanical protection. Importantly, the treatment appears to reduce muscle hyperactivity without affecting broader systemic physiological parameters or sleep architecture, indicating a favorable safety profile. For patients whose symptoms persist despite standard treatment, BTXA could be an effective adjunct to multidisciplinary management of bruxism.

## 5. Potential Mechanisms

BTXA’s neuromuscular effects may contribute to the observed reduction in bruxism activity. Toxins inhibit the release of acetylcholine at the neuromuscular junction, resulting in a temporary reduction in muscle contractility and a decrease in the amplitude of involuntary motor discharges. As a result of this mechanism, the significant decline in EMG-recorded masseter activity observed in our study is likely to be explained. In addition, some authors propose that BTXA may modulate central sensory-motor feedback loops by decreasing peripheral muscle spindle input, thus diminishing the excitability of motor pathways involved in sleep bruxism [14,16,17,18]. Despite the fact that the present study was not designed to directly evaluate central mechanisms, the substantial reduction in bruxism index observed across multiple nights supports these pathways.

## 6. Limitations of the Study

This study presents several limitations that should be considered when interpreting the findings. First, the small sample size (*n* = 10) restricts the statistical power of the analyses and limits the generalizability of the results to broader populations. The absence of a placebo or sham-injection control group further constrains the ability to distinguish the true pharmacological effect of BTX-A from potential placebo responses or natural fluctuations in bruxism activity. Additionally, the follow-up period was limited to one month, providing only short-term insight into treatment efficacy and preventing conclusions regarding the durability of therapeutic effects. In addition, potential side effects of BTX-A, including speech changes and aesthetic effects, were not systematically assessed, limiting conclusions on safety and function. Therefore, future studies should address these factors to better evaluate its therapeutic potential in sleep bruxism. Also, long-term safety outcomes could not be assessed within this timeframe, leaving unanswered questions about the sustained tolerability of repeated BTX-A administration. Finally, notable inter-individual variability in treatment response was observed, suggesting that patient-specific factors may influence therapeutic outcomes and warrant further investigation in larger, controlled studies.

## 7. Conclusions

In summary, the findings of this study indicate that intramuscular BTX-A administration to the masseter muscles resulted in a significant reduction in bruxism activity among individuals with sleep bruxism, as quantified through portable electromyographic monitoring with the Bruxoff Holter system. These results further support the potential of BTX-A as a safe and effective therapeutic approach for managing sleep bruxism. Among the secondary physiological variables, only heart rate demonstrated a meaningful post-treatment decrease, whereas respiratory rate and sleep duration showed no significant changes. Considering the limited sample size and short follow-up period, future studies should include larger cohorts of participants and extend observation periods to provide a more comprehensive assessment of long-term safety and efficacy. Furthermore, controlled trials employing placebo or sham conditions will help clarify BTX-A’s therapeutic contributions and explore the factors underlying individual variation in response to treatment.

## Figures and Tables

**Table 1:** Comparison of pre-treatment and post-treatment of patients with sleep bruxism.

Variables	Pre-Treatment	Post-Treatment	Mean Difference	Test	*p*-Value	Effect Size	Magnitude
Bruxism Index (Mean ± SD)	12.2 ± 1.32	7.4 ± 1.35	−4.8 ± 1.48	Paired	<0.001	3.25	Large
Heart rate (Mean ± SD)	64.4 ± 2.99	62.60 ± 2.63	−1.8 ± 1.99	Paired	0.018	0.91	Large
Respiratory rate (Mean ± SD)	15 ± 2.05	14.10 ± 1.85	−0.90 ± 1.37	Paired	0.068	0.66	Medium
Sleep hours (Mean ± SD)	7.2 ± 0.35	7.25 ± 0.35	0.05 ± 0.16	Wilcoxon	1.00	NA	NA

## Data Availability

The original contributions presented in this study are included in the article. Further inquiries can be directed to the corresponding author.
